# ADAMTS13 and Non-ADAMTS13 Biomarkers in Immune-Mediated Thrombotic Thrombocytopenic Purpura

**DOI:** 10.3390/jcm12196169

**Published:** 2023-09-24

**Authors:** Quintijn Bonnez, Kazuya Sakai, Karen Vanhoorelbeke

**Affiliations:** 1 Department of Chemistry, KU Leuven Campus Kulak Kortrijk, 8500 Kortrijk, Belgium; 2 Department of Blood Transfusion Medicine, Nara Medical University, Kashihara 634-8522, Japan

**Keywords:** immune-mediated thrombotic thrombocytopenic purpura, differential diagnosis, ADAMTS13 testing, ADAMTS13 activity, ADAMTS13 conformation, ADAMTS13 antigen, non-ADAMTS13 biomarkers

## Abstract

Immune-mediated thrombotic thrombocytopenic purpura (iTTP) is a rare medical emergency for which a correct and early diagnosis is essential. As a severe deficiency in A Disintegrin And Metalloproteinase with ThromboSpondin type 1 repeats, member 13 (ADAMTS13) is the underlying pathophysiology, diagnostic strategies require timely monitoring of ADAMTS13 parameters to differentiate TTP from alternative thrombotic microangiopathies (TMAs) and to guide initial patient management. Assays for conventional ADAMTS13 testing focus on the enzyme activity and presence of (inhibitory) anti-ADAMTS13 antibodies to discriminate immune-mediated TTP (iTTP) from congenital TTP and guide patient management. However, diagnosis of iTTP remains challenging when patients present borderline ADAMTS13 activity. Therefore, additional biomarkers would be helpful to support correct clinical judgment. Over the last few years, the evaluation of ADAMTS13 conformation has proven to be a valuable tool to confirm the diagnosis of acute iTTP when ADAMST13 activity is between 10 and 20%. Screening of ADAMTS13 conformation during long-term patient follow-up suggests it is a surrogate marker for undetectable antibodies. Moreover, some non-ADAMTS13 parameters gained notable interest in predicting disease outcome, proposing meticulous follow-up of iTTP patients. This review summarizes non-ADAMTS13 biomarkers for which inclusion in routine clinical testing could largely benefit differential diagnosis and follow-up of iTTP patients.

## 1. TTP: Pathophysiology, Diagnosis, Therapy and Follow-Up

### 1.1. Pathophysiology

Thrombotic thrombocytopenic purpura (TTP) is a rare disease with an incidence estimated at around four cases per million, caused by a severe deficiency of the enzyme ADAMTS13 (A Disintegrin And Metalloproteinase with ThromboSpondin type 1 repeats, member 13), and is recognized as thrombotic microangiopathy (TMA) that is characterized by severe thrombocytopenia, hemolytic anemia, and ischemic organ failure [1,2]. The substrate of ADAMTS13 is the multimerc protein von Willebrand factor (VWF). VWF is synthesized in endothelial cells and megakaryocytes, where it is stored as ultra-large prothrombotic multimers in Weibel Palade bodies and α-granules respectively. Release of the ultra-large (UL) VWF multimers results in unfolding of the VWF molecule and exposure of the ADAMTS13 cleavage site and platelet binding sites. Subsequent cleavage of UL-VWF results in smaller VWF multimers that are not prothrombotic and hence do not spontaneously bind platelets. Therefore, ADAMTS13 deficiency in TTP patients results in systemic microvascular thrombi formation, leading to platelet consumption and ischemic organ damage [3,4]. About 5% of the TTP patients suffer from the congenital form of the disease, while 95% have the acquired form of TTP. Of those, up to 90% of the TTP cases are classified as immune-mediated TTP (iTTP), resulting from the acquired production of anti-ADAMTS13 autoantibodies [5,6] while the remaining patients suffer from TTP of unknown cause (uTTP) [7,8]. 

### 1.2. Diagnosis

Historically, a clinical pentad has been used for the clinical diagnosis of TTP by screening for fever, thrombocytopenia, hemolytic anemia, and renal and neurologic dysfunction [9]. It was shown that patients with all five symptoms, only seen in 40% of cases, have poorer outcomes than those without [9]. Based on the current consensus and guidelines, TTP is clinically diagnosed by the presence of severe thrombocytopenia (<30 × 10^9^/L) and microangiopathic hemolytic anemia with highly elevated lactate dehydrogenase (LDH) and indirect bilirubin [10,11,12]. Since these signs and symptoms overlap with those of other microangiopathies, confirmation of severely reduced ADAMTS13 activity (<10%) is crucial to confirm the diagnosis of TTP. Current diagnostic assays to determine ADAMTS13 activity are based on the use of a VWF fragment, which has an exposed ADAMTS13 cleavage site. Indeed, full-length VWF adopts a folded conformation with a cryptic ADAMTS13 cleavage site and can only be used in vitro under static conditions as an ADAMTS13 substrate when it is denatured [13]. Frequently used ADAMTS13 assays include the fluorescence resonance energy transfer (FRET)-VWF73 assay and the chromogenic ADAMTS13 activity ELISA, both using an artificial 73 amino acid residue-long VWF A2 peptide (VWF73) that contains the exposed ADAMTS13 cleavage site. In the FRET-VWF73 assay, an increased fluorescence is generated when the substrate is cleaved by plasma ADAMTS13 [14]. In the chromogenic ADAMTS13 activity ELISA, the anti-N10 monoclonal antibody recognizes the digested VWF73 fragment when cleaved by ADAMTS13 [15]. Of note, the use of EDTA-treated plasma should be avoided since EDTA retrieves divalent cations from the ADAMTS13 metalloprotease domain, rendering ADAMTS13 inactive [16]. To discriminate iTTP from cTTP, the presence of autoantibodies is studied using either an anti-ADAMTS13 IgG detection ELISA or a Bethesda assay [17]. In a few iTTP cases, autoantibodies are undetectable, which is explained by the presence of immune complex formation [18]. 

### 1.3. Therapy

Standard iTTP therapy combines therapeutic plasma exchange (TPE) and corticosteroids and has significantly reduced mortality in acute-phase iTTP below 10–20% [19,20]. Recently, targeted therapies can be administered, including anti-CD20 monoclonal antibody (rituximab) [21,22,23] and anti-VWF A1 nanobody (caplacizumab) [24,25], and these therapies enable successful treatment of iTTP patients in the acute phase. However, prophylactic treatment options to prevent iTTP relapse during remission remain urgent since 20–50% of patients experience at least one clinical relapse [26,27,28,29]. Standard cTTP therapy includes plasma infusions until TTP-related symptoms have resolved and platelet counts have normalized. Prophylactic plasma infusions are given in the remission phase to prevent the recurrence of cTTP [2,8].

### 1.4. Follow-Up

During remission, treating physicians typically provide monthly follow-up for the first three months, three-monthly follow-up for the first year, and six-monthly or yearly follow-up when stable [30]. Platelet counts, LDH levels, ADAMTS13 activity, and inhibitory and non-inhibitory ADAMTS13 antibodies should be carefully followed during each visit to anticipate an ADAMTS13 or clinical relapse in the super early stage and to treat the patients accordingly. Nonetheless, accurately identifying patients at risk of relapse in iTTP remains a significant challenge due to the absence of reliable biomarkers. However, numerous studies have focused on assessing the prognostic potential of various biomarkers in order to enhance the prediction of relapse. In the next section, these different biomarkers and their role in predicting an iTTP relapse will be discussed.

## 2. ADAMTS13 Antigen, Autoantibodies and Conformation to Advance Diagnosis and Follow-Up

### 2.1. ADAMTS13 Activity, Antigen and Autoantibodies

#### 2.1.1. Low ADAMTS13 Activity and/or Presence of Anti-ADAMTS13 IgG and Their Link with Relapse

When iTTP patients suffer from clinical relapse, ADAMTS13 tests reveal severely deficient ADAMTS13 activity and positive anti-ADAMTS13 IgG as laboratory findings. Persistent or recurrent deficiency of ADAMTS13 activity during follow-up of survivors of acute iTTP is an established risk factor for disease recurrence [27,31]. Accordingly, many groups have discussed the importance of monitoring ADAMTS13 activity and anti-ADAMTS13 IgG titers in the follow-up. Ferrari et al. reported a prospective cohort study that enrolled 35 iTTP patients during an 18-month follow-up [26]. Occurring on one or two occasions (19%) throughout the follow-up period, elevated levels of inhibitory anti-ADAMTS13 IgG upon initial presentation were linked to the continuous absence of detectable ADAMTS13 activity during remission. Among 13 survivors with undetectable ADAMTS13 activity in remission, six experienced a relapse. Hence, depleted ADAMTS13 activity with detectable autoantibodies was indicative of forthcoming relapses within an 18-month timeframe [26]. Peyvandi et al. also confirmed these findings in a retrospective iTTP cohort; severe deficiency of ADAMTS13 activity and positive anti-ADAMTS13 antibodies were more identified in patients with recurrent iTTP relapses than those without relapses [27]. In addition, the likelihood of relapse was 3.6 times higher when patients had both severe ADAMTS13 deficiency and anti-ADAMTS13 antibodies [27]. Using a logistic regression model, Jin et al. assessed the relationship between ADAMTS13 activity level and the probability of iTTP relapse in 157 serial samples from 24 patients [31]. The authors revealed that lower ADAMTS13 activity and younger age were significantly linked to a higher risk of relapse three months after sample withdrawal, whereas ADAMTS13 antibody IgG levels were not predictive of iTTP relapses [31]. Schieppati et al. revealed in a multi-institutional study that the correlation between ADAMTS13 activity being ≤20% and a significant anti-ADAMTS13 titer during remission, along with a duration of at least 13 days for the initial treatment’s response, were autonomous prognostic indicators for the recurrence of the disease [32].

As for ADAMTS13 parameters in acute phase, Sui et al. described that while plasma levels of ADAMTS13 activity, antigen, and anti-ADAMTS13 IgG on admission could not predict exacerbation or recurrence in patients with iTTP, persistently low plasma ADAMTS13 activity below 10 U/dL (HR, 4.4; *p* < 0.005) or high anti-ADAMTS13 IgG (HR, 3.1; *p* < 0.016) 3 to 7 days after the initiation of TPE was associated with a higher risk for exacerbation or recurrence [33].

In conclusion, persistently depleted ADAMTS13 activity accompanied by positive ADAMTS13 autoantibodies in the middle of the acute phase and during remission causes earlier iTTP relapse.

#### 2.1.2. Low ADAMTS13 Antigen and High Anti-ADAMTS13 IgG and Their Link with Disease Outcome and Prognosis

To date, the ADAMTS13 antigen is not routinely evaluated in clinical practice. In healthy individuals, normal ADAMTS13 antigen levels are found to range between 0.5 and 1.8 µg/mL [34,35,36,37]. Although iTTP patients sporadically display ADAMTS13 antigen within the normal range, almost all patients present with severely deficient antigen levels during acute phase iTTP [34,35,36]. ADAMTS13 antigen depletion at presentation is statistically associated with disease severity, as significantly lower presenting antigen levels are detected in patients with fatal disease outcomes, and a five-fold higher mortality rate is associated with ADAMTS13 antigen levels in the lowest quartile [34,35]. Therefore, ADAMTS13 antigen levels could serve as a prognostic factor to predict disease outcome. Follow-up of ADAMTS13 antigen during treatment could also provide helpful information to guide patient management, as higher antigen levels at clinical response suggest patients should sustain remission [36]. Intriguingly, iTTP patients displaying no inhibitory antibodies were found to have significantly lower ADAMTS13 antigen levels at first presentation [34]. However, when inhibitory antibodies are detected, lower antigen levels are observed in patients with autoantibodies against both N- and C-terminal ADAMTS13 domains compared to patients with only N-terminal antibodies [34]. 

Despite the polyclonal immune response in iTTP, dominant immunoprofiles suggest that nearly all patients display antibodies targeted against immunogenic hotspots mainly located in the ADAMTS13 Spacer (S) and Cysteine-rich (C) domains (hereafter referred to as anti-CS antibodies) [34,38,39,40,41,42]. However, the domain specificity of presenting anti-ADAMTS13 antibodies does not differ between surviving and deceased iTTP patients. Moreover, disease severity, prognosis, or patient management to enable remission could not be linked to antibody domain specificity nor to the three most dominant patient immunoprofiles [34,39,42]. Aberrantly high antibody titers, typically found when multiple domains are targeted by anti-ADAMTS13 antibodies [42], are displayed in over 90% of presenting patients, even though elevated titers could often only be measured at the later stages of relapse episodes [35]. Interestingly, patients with antibody titers in the highest quartile showed a three-fold increased mortality rate when compared to those in the lowest quartile. Additionally, elevated troponin levels, a lowered Glasgow Coma Scale (GCS) score, and a larger number of plasma exchange sessions were associated with patients in the highest quartile [35]. Therefore, both ADAMTS13 antibody (i.e., titers of the highest quartile) and antigen (i.e., titers of the lowest quartile) are reported to adversely affect TTP outcome by means of elevated mortality rates as well as raised cardiac and neurological involvement [34,35,42]. 

### 2.2. Open ADAMTS13 Conformation and Its Link with Diagnosis and Follow-Up

As previously described, the diagnosis of iTTP is always confirmed when the laboratory parameters show an ADAMTS13 activity below 10% and the presence of anti-ADAMTS13 antibodies [2,10,42]. However, diagnosis remains challenging when borderline ADAMTS13 activity levels fluctuate between 10–20%, as alternative thrombotic microangiopathies could be differentially diagnosed [11,42]. To correctly diagnose such iTTP patients, clinical attention is attracted towards a novel biomarker: an open ADAMTS13 conformation, as conformationally altered self-antigens are also observed in other autoimmune diseases [42,43,44,45,46,47]. Indeed, our group showed that an open ADAMTS13 conformation is a specific biomarker for acute iTTP as well as for subclinical iTTP [43,44]. In acute iTTP patients, the ADAMTS13 conformation is open, whereas the evaluation of healthy individuals, sepsis patients, and hemolytic uremic syndrome (HUS) patients all showed a closed conformation [43]. This suggests successful differentiation of acute iTTP patients from patients with alternative TMAs such as HUS, which could thereby largely benefit the diagnosis of iTTP patients that present ADAMTS13 activity ranging from 10 to 20%. Long-term follow-up of individual iTTP patients revealed that nearly all remission patients with ADAMTS13 activity below 50% had an ADAMTS13 with an open ADAMTS13 conformation, demonstrating that open ADAMTS13 is not only a biomarker for acute iTTP but also for subclinical disease. Intriguingly, ADAMTS13 conformation was closed in over 60% of remission patients with >50% ADAMTS13 activity, indicating that open ADAMTS13 might predict relapse in these patients [44]. 

We showed that iTTP patient anti-ADAMTS13 antibodies induce an open ADAMTS13 conformation. Indeed, purified iTTP patient IgG and, more specifically, purified anti-CS antibodies induced an open ADAMTS13 conformation [47]. This finding linked the open ADAMTS13 conformation with the most dominant immunoprofile (i.e., presence of only anti-CS antibodies) described in both Caucasian and Japanese iTTP patient cohorts [34,39,42,47]. On the other hand, purification of patient anti-CUB antibodies, present in over 50% of patients [39,42], revealed that only some of these anti-CUB fractions could induce an open ADAMTS13 conformation. This observation might explain how ADAMTS13 could adopt an open conformation in iTTP patients without detectable anti-CS antibodies [47]. The role of anti-ADAMTS13 antibodies in opening ADAMTS13 is in line with the observation that in acquired TTP of unknown pathophysiology (uTTP), a closed ADAMTS13 conformation is typically presented by such patients (>85%), and no anti-ADAMTS13 antibodies are detected [48]. The role of anti-ADAMTS13 antibodies in opening ADAMTS13 was also confirmed by the observed changes in ADAMTS13 conformations when preemptive rituximab administration was used. Patients responsive to rituximab treatment (i.e., >50% ADAMTS13 activity recovery) systematically recovered a closed ADAMTS13 conformation; however, a borderline open conformation was also reported in some patients [44,49]. Alternatively, an open ADAMTS13 conformation was sustained in patients poorly responding to preemptive rituximab, as no ADAMTS13 activity restoration or anti-ADAMTS13 antibody titer reduction occurred [49]. 

Finally, although ADAMTS13 activity was decreased in the remission patients with an activity <50%, anti-ADAMTS13 antibodies were often undetectable [44,49,50]. Since we showed that iTTP patient anti-ADAMTS13 antibodies induce an open ADAMTS13 conformation, open ADAMTS13 could be a surrogate marker for the presence of anti-ADAMTS13 antibodies when these antibodies are undetectable in the plasma of these patients. As ELISA-assays used to detect anti-ADAMTS13 antibodies in patient plasma typically only identify free antibodies, undetectable antibody levels can be explained by their presence in immune complexes. On the other hand, very low levels of free antibodies might be undetectable in the current ELISAs [18,44,51,52]. 

To date, the reference assay to evaluate ADAMTS13 conformation is the 1C4 open/closed ELISA developed in our group, in which a cryptic epitope in the Spacer domain of open ADAMTS13 is specifically recognized [42,43,44,47]. Novel diagnostic tests to evaluate ADAMTS13 conformation that are fast, automated, and easy-to-use could be beneficial to promote iTTP diagnosis and ameliorate patient follow-up and management.

## 3. Non-ADAMTS13 Parameters

### 3.1. Troponin-T/I and Glasgow Coma Score and Their Link with Acute iTTP Death

Cardiac troponin-T and-I (cTnT and cTnI) are biomarkers commonly used for detecting myocardial injury and the differential diagnosis of acute coronary syndrome [53,54]. Patients with acute decompensated heart failure with positive cardiac troponin tests had lower systolic blood pressure on admission, a lower ejection fraction, and higher in-hospital mortality than those with negative tests [55]. In addition, patients with a positive troponin are 2.55 times more likely to die than those not positive for troponin (95% confidence interval, 2.24 to 2.89; *p* < 0.001 by the Wald test) [55]. Although induction of plasma exchange has dramatically improved the survival rate in acute iTTP, the 30-day mortality rate remains 10–20% [19,20]. Acute cardiac events in iTTP are myocardial infarction, congestive heart failure, fatal arrhythmias, and cardiogenic shock, leading to fatal outcomes in the acute phase [56]. A Japanese retrospective study revealed that 26 out of 32 patients experienced sudden death, mostly following radical hypotension and bradycardia. The median follow-up time after admission was 5.0 days, and nine patients underwent autopsy and had cardiac microvascular thrombi in arterioles [57]. The UK group reported a retrospective study on cardiac involvement in acute iTTP. A positive cTnT test was identified in 54% of patients, and half had cardiac symptoms [58]. Intriguingly, an elevated anti-ADAMTS13 IgG titer was associated with positive cTnT above 0.25 ng/mL (normal range 0–0.01 ng/mL), and both parameters predicted mortality and acute morbidity in acute iTTP [58]. Moreover, cTnI in iTTP was also assessed by the French TMA reference center [59]. An increased cTnl above 0.1 ng/mL was seen in 78 out of 133 non-selected patients, of whom 46 had no clinical cardiac involvement. A cTnl level of >0.25 ng/mL was determined as an independent predictive factor in death and refractoriness (odds ratio 2.87 and 3.03, respectively) [59]. Based on these studies, elevated cTnT/cTnI on admission would predict poor clinical outcomes, probably because patients with higher cTnT/cTnI substantially suffer from a cardiac injury due to microthrombi [58,59]. However, it should be noted that not all patients with positive cTnT/cTnI develop cardiac involvement in the acute phase [58,59]. 

The GCS is a clinical scale used to reliably measure a person’s level of consciousness after a brain injury. Its score is based on eye opening (ocular response, 1–4), verbal (oral response, 1–5), and motor responses (motoric response, 1–6). The combined score, which ranges from 3 to 15, reflects consciousness. Generally, brain injury is classified as Severe, GCS ≤ 8; Moderate, GCS 9–12; or Minor, GCS ≥ 13 [60]. Alwan et al. reported that 24% of iTTP patients had a reduced GCS, defined as a GCS score of 14 or below, at presentation, with a ninefold increase in mortality (20% vs. 2.2% for normal GCS at presentation, *p* < 0.0001) [35]. In this study, while cardiac involvement was also identified as a risk for mortality, there was no synergistic effect on the mortality rate of a combined decreased GCS and elevated cardiac troponin compared with the mortality for a single abnormal prognostic factor [35,61]. A further prospective investigation is required to conclude if a novel anti-VWF A1 nanobody, caplacizumab, could improve patients with positive cTnT/cTnI and/or impaired GCS.

### 3.2. Markers of Endothelial Activation and Inflammation

Endothelial cell (EC) activation and inflammation have been linked to the pathophysiology of iTTP [62,63,64,65,66]. Hence, proteins secreted from endothelial cells and/or circulating endothelial cells (CECs) or proteins secreted from leukocytes during acute iTTP and in remission might be interesting biomarkers to predict disease outcome and relapse. In this section, proteins and cells that have been studied as possible biomarkers for disease outcome will be discussed.

Although VWF antigen is increased during the acute phase upon EC activation, high VWF antigen levels were not predictive of disease outcome or relapse [27,67]. In contrast, decreased high molecular weight (HMW) VWF multimers were associated with the presence and severity of neurological symptoms in acute phase iTTP, while no association was found between HMW VWF and relapse in the French retrospective cohort study [68]. In the Mainz prospective cohort study, a newly defined fraction of HMW compared to LMW VWF multimers (VWF MM ratio) was shown to be higher in patient plasma samples obtained a few days to several weeks before a relapse compared to patients remaining in remission [69]. Whether changes in HMW VWF multimers during remission predict relapse remains to be determined. Moreover, soluble P-selectin (sP-selectin) concentration is elevated in the acute phase upon EC activation; however, increased sP-selectin concentrations were not associated with neurological symptoms nor with disease severity [66]. 

Another protein secreted upon EC activation is big endothelin-1 (Big ET-1). Big ET-1 is a 38-amino acid polypeptide and the precursor of ET-1, a potent vasoconstrictor. The half-life of ET-1 is less than one minute, while Big ET-1 is more slowly cleared. Big ET-1 is synthesized in vascular ECs, where it has been identified in Weibel Palade bodies. It was shown that plasma levels of Big ET-1 are significantly elevated upon admission and during clinical response/remission [65]. Elevated levels of plasma Big ET-1 upon admission were linked to acute renal insufficiency and higher in-hospital mortality rates. Furthermore, persistently elevated plasma levels of Big ET-1 during clinical response/remission are associated with exacerbations of iTTP. Whether plasma levels of Big ET-1 were associated with the risk of relapse was not investigated [65]. A possible role of Big ET-1 in the pathophysiology of iTTP is not known.

Finally, CECs, which are stressed endothelial cells that become detached from the endothelial membrane and indicate endothelial damage, were elevated during acute phase iTTP. This increase was linked to the presence of initial neurological symptoms and demonstrated a correlation with the patient’s clinical outcome. Whether an increase in CECs predicts relapse remains to be determined [66]. 

Syndecan-1 (Sdc-1) and soluble thrombomodulin (sTM) are the main components of the endothelial glycocalyx, a layer of membrane-bound macromolecules anchored to the luminal surface of the vascular endothelium. In specific pathologic conditions, such as acute inflammation and ischemia-reperfusion injury [70,71,72], leukocyte-derived proteases, metalloproteinases, and heparinases cleave the ectodomains of Sdc-1 and TM. Upon admission, individuals with acute iTTP exhibit significantly higher plasma levels of Sdc-1 and/or sTM, and these levels remain elevated during clinical response/remission. Increased plasma levels of Sdc-1 and/or sTM on admission are linked to mortality in patients experiencing acute iTTP. A concurrent rise in plasma Sdc-1 and sTM during clinical response/remission is associated with a higher recurrence rate of acute iTTP [64].

Granular, azurophilic neutrophil content, such as S100A8/A9, human neutrophil peptides 1–3 (HNP1–3), and neutrophil extracellular traps (NETs), are released upon inflammation or neutrophil activation [62]. Moreover, neutrophils, as a NET component, as well as different cell processes such as necrosis and apoptosis, cause the release of cell-free DNA (cfDNA). And as NETs consist of neutrophil proteases and histone/DNA complexes, increased levels of these plasma markers (i.e., cfDNA, S100A8/A9, and histone/DNA complexes) are reported in acute iTTP patients. Notably, elevated levels of these plasma markers at admission are associated with in-hospital patient mortality [62]. Interestingly, HNP1-3 is described to bind the VWF A2 domain, which could thereby inhibit multimeric VWF cleavage by ADAMTS13. Intriguingly, HNP1–3 share a RRY peptide motif with the immunogenic ADAMTS13 spacer domain, which might suggest that HNPs could enhance pathogenic autoantibody production [73].

Markers of endothelial and leukocyte activation are increased in acute iTTP, and some of these are associated with neurological symptoms or patient outcomes. However, larger prospective studies are needed to prove their usefulness in predicting disease outcome and relapse.

## 4. Conclusions

**Figure 1:**
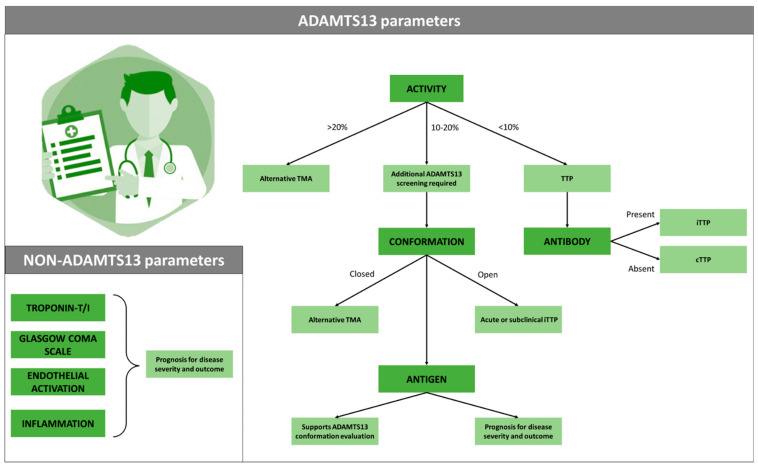
(Non)-ADAMTS13 diagnostic parameters for iTTP. To diagnose TTP, evaluation of different ADAMTS13 parameters is paramount. ADAMTS13 activity levels below 10% of normal activity specifically discriminate TTP from alternative TMAs. Diagnosis remains challenging when an activity between 10 and 20% is presented, requiring additional ADAMTS13 testing to correctly recognize TTP. The presence of anti-ADAMTS13 antibodies suggests the subdiagnosis of iTTP, whereas its absence could indicate cTTP. Within this 10–20% activity range, the ADAMTS13 conformation, relying on ADAMTS13 antigen evaluation, provides crucial information to properly (sub)diagnose TTP disease. Anyway, its contribution to conformation determination, the ADAMTS13 antigen is described as a prognostic factor for disease severity and clinical outcome. Non-ADAMTS13 parameters (troponin levels, GCS, endothelin-1, histone/DNA complexes, and syndecan-1) lack specificity to diagnose TTP, despite being described as valuable tools to predict disease outcome and guide patient management. TMA, thrombotic microangiopathy; ADAMTS13 (A Disintegrin And Metalloproteinase with ThromboSpondin type 1 Repeats, Member 13); TTP, thrombotic thrombocytopenic purpura; iTTP, immune-mediated TTP; cTTP, congenital TTP.

This review summarizes essential biomarkers to differentially diagnose iTTP from alternative TMAs and allow subdiagnosis of various iTTP forms (Figure 1). Clinical evaluation of ADAMTS13 parameters (activity, antibody, conformation, and antigen) is essential to specifically diagnose iTTP, with an open ADAMTS13 conformation serving as a sensitive tool to confirm iTTP when ADAMTS13 activity ranges between 10 and 20%, even when anti-ADAMTS13 antibodies remain undetectable. ADAMTS13 antigen levels as well as some non-ADAMTS13 parameters could be assessed to predict iTTP disease severity and mortality. To date, the role of each ADAMTS13 parameter for clinical diagnosis and prognosis of iTTP patients has been thoroughly evaluated, whereas the role of various non-ADAMTS13 biomarkers remains indefinite. Accurate and easily available tests are prerequisites, and additional clinical studies are needed to clarify the potential role of each of these non-ADAMTS13 parameters in iTTP diagnosis and prognosis. Therefore, the inclusion of automated, easy-to-use assays for these novel biomarkers in routine clinical testing might largely benefit on-demand diagnosis and follow-up of iTTP patients while providing essential insights into disease progression and allowing rapid switching of treatment administration.
